# The Effect on the Kidney in Patients With Anti-N-methyl D-aspartate Receptor Antibody Encephalitis

**DOI:** 10.3389/fneur.2021.601495

**Published:** 2021-02-12

**Authors:** Lizhi Liu, Meifeng Gu, Jia Liu, Qing Liu, Xiaofeng Xu, Rong Fan, Fuhua Peng, Ying Jiang

**Affiliations:** ^1^Department of Neurology, The Third Affiliated Hospital, Sun Yat-sen University, Guangzhou, China; ^2^Department of Neurology, Huizhou Hospital of Traditional Chinese Medicine, Huizhou, China

**Keywords:** anti-N-methyl-D-aspartate receptor encephalitis, estimated glomerular filtration rate (eGFR), renal function, urinalysis, urine pH, urine specific gravity (USG)

## Abstract

**Objectives:** The function of the N-methyl-D-aspartate receptor (NMDAR) in the kidney has been studied. However, the effect on the kidney from anti-NAMDAR antibody encephalitis has not been investigated thus far.

**Methods:** Case data were collected from 82 patients with anti-NMDAR antibody encephalitis and 166 age- and sex-matched healthy controls (HCs). Clinical characteristics, urinalysis [including urine pH and urine specific gravity (SG)], serum creatinine (Scr), and estimated glomerular filtration rate (eGFR) based on Cr levels were evaluated.

**Results:** At initial admission, urine pH levels and urine SG levels in anti-NMDAR antibody encephalitis patients were significantly higher and lower, respectively, than HCs (both *p* < 0.001). There were no significant differences in Scr and eGFR between anti-NMDAR antibody encephalitis patients and HCs. Urine pH levels in patients with anti-NMDAR antibody <1:32 were significantly lower than those in patients with anti-NMDAR antibody ≥1:32 (*p* = 0.029). Urine pH levels were significantly lower (*p* = 0.004) and urine SG levels were significantly higher (*p* = 0.027) in a follow-up evaluation 3 months after treatment.

**Conclusions:** The changes in urinalysis occur in patients with anti-NMDAR antibody encephalitis. The pathophysiological changes in anti-NMDAR antibody encephalitis were not limited to the CNS.

## Introduction

The anti-N-methyl-D-aspartate receptor (anti-NMDAR) antibody encephalitis is the most common antibody-mediated encephalitis (1) and is caused by the production of autoantibodies against the GluN1 subunit of NMDAR (2). It represents a severe neuropsychiatric manifestation characterized by seizures, memory decline, and behavioral deficits (3, 4).

Microarray studies haveshown that all known NMDAR transcripts can be detected in the kidney (5), and there is now a consensus that activation of these receptors affects renal function, and in some cases may induce renal dysfunction (6). NMDARs are expressed in the renal cortex and medulla and appear to play a role in the regulation of renal blood flow, glomerular filtration, proximal tubule reabsorption, and urine concentration within medullary collecting ducts (6).

Glomerular filtration, tubular reabsorption, and tubular excretion are three mechanisms through which kidneys accomplish the homeostasis of the internal environment (7). The glomerular filtration rate (GFR) is a universal marker of renal function (8) and classically used for evaluating individual's kidney function and for scoring disease stages in chronic kidney disease (CKD) patients (9). Urine specific gravity (SG) correlates with urine osmolality and reflects the concentrating ability of the kidneys (10). Urine pH is generally used to provide an overall estimate of a patient's acid-base status and reflects the pH of body fluids (11). In patients with anti-NMDAR antibody encephalitis, it is not clear whether anti-NMDAR antibodies could lead to damage in the kidney resulting in abnormal urinalysis and renal function.

To the best of our knowledge, no studies have been conducted to analyze the urinalysis and renal function in patients with anti-NMDAR antibody encephalitis. The objective of this study is to evaluate the results from urinalysis and renal function between anti-NMDAR antibody encephalitis and HCs, including urine SG, pH, serum creatinine (SCr), and estimated GFR (eGFR).

## Methods

### Study Design and Samples

This study is approved by the Medical Ethics Committee of the Third Affiliated Hospital of Sun Yat-sen University. All study participants have provided written consent for research and publication.

We recruited 82 Chinese Han patients with anti-NMDAR antibody encephalitis from the Department of Neurology at the Third Affiliated Hospital of Sun Yat-sen University during March 2015 to November 2019. Diagnosis criteria for anti-NMDAR antibody encephalitis were based on the diagnostic criteria by Graus et al. (12): (1). The presence of one or more of the six major groups of symptoms: (i) Abnormal (psychiatric) behavior or cognitive dysfunction; (ii) Speech dysfunction (pressured speech, verbal reduction, mutism); (iii) Seizures; (iv) Movement disorder, dyskinesias, or rigidity/abnormal postures; (v) Decreased level of consciousness; and (vi) Autonomic dysfunction or central hypoventilation. (2). Anti-NMDAR antibody testing in cerebrospinal fluid (CSF) was positive. (3). Reasonable exclusion of other disorders. Additionally, none of the patients had urinary tract infections, stones in the kidney or urinary tract, or obviously abnormal thyroid function at initial admission or at follow-up. Anti-NMDAR antibodies in CSF, or both CSF and serum, were investigated with a cultured cell-based method using a commercially available kit (EUROIMMUN Medizinische Labordiagnostika, Lübeck, Germany) according to the manufacturer's instructions. All patients were clinically evaluated for neurological status using the modified Rankin Scale (mRS) scores (13) and screened for systemic tumors with computed tomography (CT), positron emission tomography-computed tomography (PET-CT), magnetic resonance imaging (MRI), or B ultrasound at least once. We also recruited 166 healthy controls (HCs) age- and sex-matched to anti-NMDAR antibody encephalitis patients from the Department of Medical Examination Center.

### Urinalysis and Renal Function Assessment at Initial Admission

Early morning spot urine samples were collected from all anti-NMDAR antibody encephalitis patients on the day after admission to measure urine SG and pH. After urine samples were collected at initial admission, all anti-NMDAR antibody encephalitis patients received methylprednisolone pulse therapy or intravenous immunoglobulin.

Serum Cr levels were also measured in all anti-NMDAR antibody encephalitis patients on the day after admission. The eGFR based on Cr was calculated using the Chronic Kidney Disease Epidemiology Collaboration (CKD-EPI) formula (14).

### Follow-Up Evaluations

Among these 82 anti-NMDAR antibody encephalitis patients, 32 patients had a follow-up evaluation 3 months after treatment. The follow-up evaluation included the repetition of mRS scores and the measurement of serum Cr, eGFR, urine SG, and urine pH.

### Statistical Analysis

All statistical analyses were performed using the Statistical Program for Social Sciences (SPSS) software (version 22.0, Chicago, IL, USA). The data were presented as mean ± standard deviation (SD) if the data was normally distributed or as median and interquartile range (IQR) if the data was not normally distributed. Unless otherwise noted, we used student *t*-test for testing the difference of normally distributed variable from two groups, Mann-Whitney *U*-test (also known as Wilcoxon rank-sum test) for testing the difference of non-normally distributed variable from two groups, and Chi-square test for testing the association of two binary variables. Paired *t*-test was used for normal data and paired Mann-Whitney *U*-test was used for non-normal data. One-way analysis of variance (ANOVA) and the Scheffe *post-hoc* test were used for statistical comparisons among the HC, acute phase, and stable phase groups. To eliminate the effects of age and sex, a multivariable linear regression model was used to determine the differences in the urine pH levels and urine SG levels among the groups. All tests were two-sided with a significance level of 0.05.

## Results

### Baseline Characteristics

Table 1 shows the baseline characteristics between anti-NMDAR antibody encephalitis patients (female: male = 37:45) and HCs (female: male = 76:90). The median mRS score and disease duration in anti-NMDAR antibody encephalitis patients were 4 (1.75–5.00) and 28.63 ± 19.66 days, respectively. 48 of 82 patients (58.54%) had seizures. 14 of 82 patients (17.07%) had tumors.

**Table 1:** Demographic features of patients with anti-NMDAR antibody encephalitis at initial admission and healthy controls.

	**Anti-NMDAR encephalitis (*n* = 82)**	**Healthy controls (*n =* 166)**	***p*-value**
Age onset (y, mean ± SD)	32.11 ± 12.27	31.78 ± 12.35	0.842^P1^
Gender (male: female)	37:45	76:90	1.00^P3^
Disease duration (d, mean ± SD)	28.63 ± 19.66	–	–
CSF anti-NMDAR Abs positive (*n*, %)	82 (100)	–	
Urine^*^			
Urine pH levels	7.00 (6.50–7.00)	6.00 (5.50–6.50)	<0.001^P2^
Adjusted urine pH levels	6.78 (6.75–6.90)	6.16 (5.98–6.19)	<0.001^P2^
Urine SG levels	1.020 (1.015–1.020)	1.020 (1.020–1.025)	<0.001^P2^
Adjusted urine SG levels	1.017 (1.017–1.018)	1.021 (1.020–1.023)	<0.001 ^P2^
Scr (μmol/L, IQR)	56.00 (47.75–73.25)	58.00 (51.75–68.25)	0.897^P2^
Adjusted Scr (μmol/L, IQR)	57.80 (55.31–72.70)	53.59 (51.34–68.60)	0.111^P2^
eGFR (IQR)	123.04 (108.62–133.51)	123.60 (117.82–128.81)	0.936^P2^
Adjusted eGFR	123.18 (113.08–131.64)	124.80 (118.49–128.05)	0.746^P2^
mRS (IQR)	4.00 (1.75–5.00)	0.00 (0.00–0.00)	<0.001^P2^
With seizure (*n*, %)	48 (58.54)	0 (0.00)	<0.001^P3^
With tumor (*n*, %)	14 (17.07)	–	

*anti-NMDAR, anti-N-Methyl-D-aspartate receptor; CSF, cerebrospinal fluid; Ab, antibody; Scr, Serum Creatinine; GFR, glomerular filtration rate; eGFR, estimated GFR; SG, Specific Gravity; SD, standard deviation; IQR, interquartile range*.

* *, Anti-NMDAR encephalitis n = 79 (male: female 37:42, age 32.33 ± 12.32); p1, the Student's t-test; p2, Mann-Whitney U-tests; p3, Chi-square test*.

### Comparison Between Anti-NMDAR Antibody Encephalitis Patients and Healthy Controls

Urine SG, urine pH, serum Scr, and eGFR levels were compared between anti-NMDAR antibody encephalitis patients and HCs. The results were shown in Table 1.

**Figure 1:**
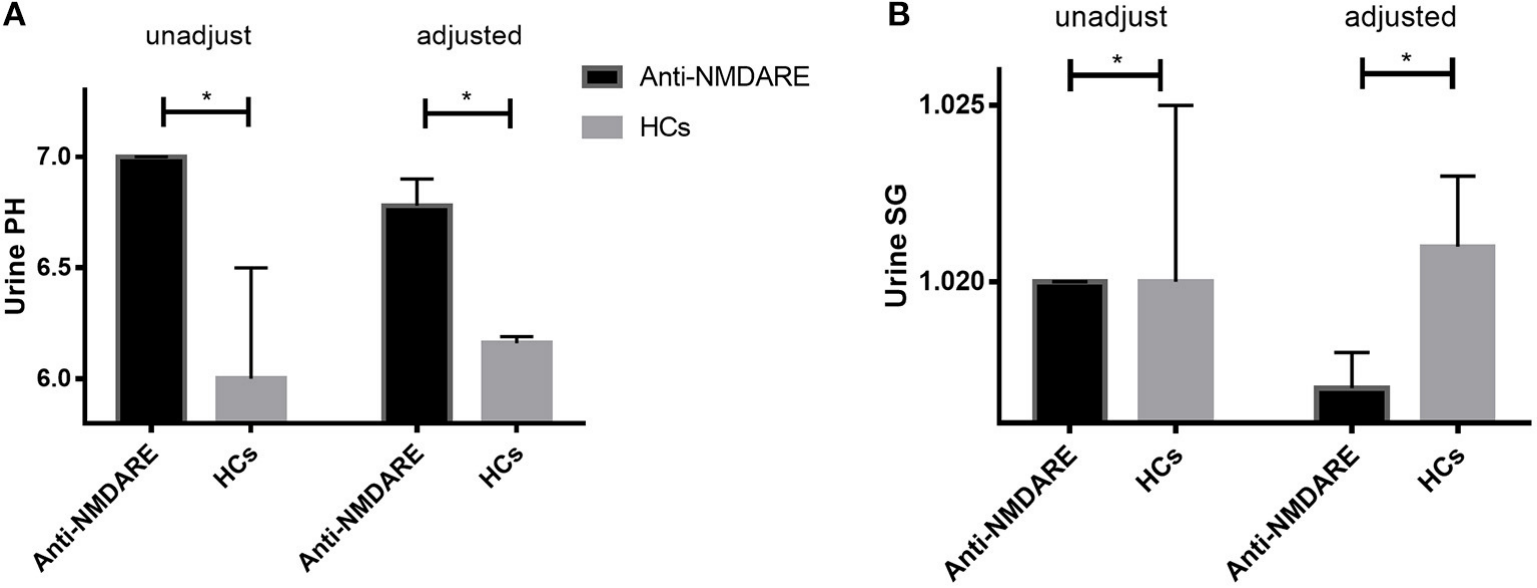
**(A)** The unadjusted and adjusted urine SG level in anti-NMDAR antibody encephalitis patients were significantly lower than that of HCs (both *p* < 0.001). **(B)** The unadjusted and adjusted urine pH levels in anti-NMDAR antibody encephalitis patients were significantly higher than HCs (both *p* < 0.001). * represents significant difference between the two groups, *p* < 0.05.

The median unadjusted and adjusted urine SG level in anti-NMDAR antibody encephalitis patients were significantly lower than median urine SG level in HCs (both *p* < 0.001; Table 1, Figure 1A). The unadjusted median and interquartile range (IQR) urine SG level were 1.020 (1.015–1.020) vs. 1.020 (1.020–1.025) and the adjusted median (IQR) were 1.017(1.017–1.018) vs. 1.021 (1.020–1.023). In both the unadjusted and adjusted models, the median (IQR) urine pH levels in anti-NMDAR antibody encephalitis patients were significantly higher than HCs (both *p* < 0.001; Table 1, Figure 1B). The unadjusted median (IQR) were 7.00 (6.50–7.00) vs. 6.00 (5.50–6.50) and the adjusted median (IQR) were 6.78 (6.75–6.90) vs. 6.16 (5.98–6.19). In addition, Scr levels in anti-NMDAR antibody encephalitis patients were also higher than HCs in the adjusted model, but with no significant differences (*p* = 0.111; Table 1). There were not any statistically significant differences in eGFR (both unadjusted and adjusted) between anti-NMDAR antibody encephalitis patients and HCs (Table 1).

### Comparison Between Anti-NMDAR Antibody <1:32 and Anti-NMDAR Antibody ≥1:32 in Anti-NMDAR Antibody Encephalitis Patients at Initial Admission

We further divided these patients into two groups according to the titers of CSF IgG antibody against NMDAR, anti-NMDAR antibody <1:32, and anti-NMDAR antibody ≥1:32. The results were shown in Table 2.

**Table 2:** Comparison between NMDAR <1:32 and NMDAR ≥1:32 in anti-NMDAR antibody encephalitis patients at initial admission.

	**NMDAR <1:32**	**NMDAR ≥1:32**	**HCs**				
**Variables**	**(*n* = 26)**	**(*n* = 48)**	**(*n =* 166)**	**p 1 vs. 2 vs. 3**	***p*-value 1 vs. 2**	***p*-value 1 vs. 3**	***p*-value 2 vs. 3**
Age onset (y, mean ± SD)	31.85 ± 12.07	30.94 ± 12.02	31.78 ± 12.35	0.894^P2^	0.757^P1^	0.979^P1^	0.746^P1^
Sex, male: female	11: 15	21: 28	76:90	0.929^P3^	0.963^P3^	0.905^P3^	0.933^P3^
Disease duration (d, IQR)	24.00 (13.00–28.25)	24.00 (15.50–38.50)	–	–	0.155^P2^	–	–
Cr (μmol/L, IQR)	61.00 (49.00–74.50)	54.00 (46.00–75.00)	58.00 (51.75–68.25)	0.874^P2^	0.628^P2^	0.596^P2^	0.932^P2^
eGFR (ml/(min × 1.73m2), IQR)	121.83(109.71–132.39)	122.64(106.90–138.22)	123.60 (117.82–128.81)	0.956^P2^	0.717^P2^	0.804^P2^	0.864^P2^
mRS	2.00 (0.75–4.00)	4.00 (2.00–5.00)	–	–	0.032^P2^	–	–
USG	1.020 (1.011–1.024)^a^	1.020 (1.015–1.020)^b^	1.020 (1.020–1.025)	<0.001^P2^	0.734^P2^	0.006^P2^	<0.001^P2^
pH	6.75 (6.00–7.00)^a^	7.00 (6.50–7.00)^b^	6.00 (5.50–6.50)	<0.001^P2^	0.029^P2^	<0.001^P2^	<0.001^P2^

*anti-NMDAR, anti-N-Methyl-D-aspartate receptor; CSF, cerebrospinal fluid; Ab, antibody; Scr, Serum Creatinine; GFR, glomerular filtration rate; eGFR, estimated GFR; SG, Specific Gravity; SD, standard deviation; IQR, interquartile range. p1, the Student's t-test; p2, Mann-Whitney U-tests; p3, Chi-square test, a, n = 24, b, n = 47. 1 refers to NMDAR <1:32; 2 refers to NMDAR ≥1:32; 3 refers to HCs*.

**Figure 2:**
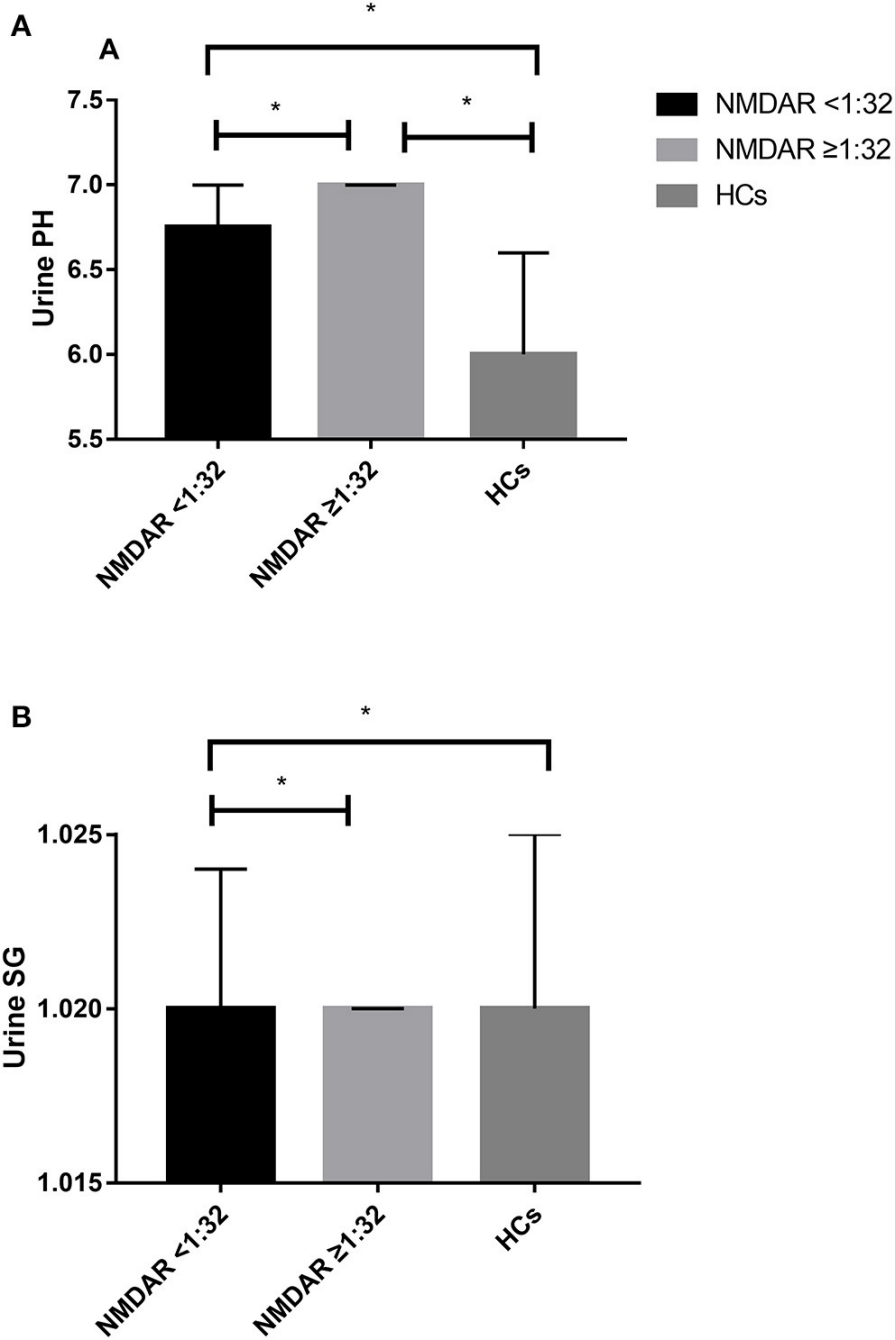
**(A)** The unadjusted median and interquartile range (IQR) urine pH levels in patients with anti-NMDAR antibody <1:32 were significantly lower than those in patients with anti-NMDAR antibody ≥1:32 (*p* = 0.029). The median (IQR) urine pH levels in anti-NMDAR antibody encephalitis patients with anti-NMDAR antibody <1:32 and anti-NMDAR antibody ≥1:32 were significantly higher than HCs (both *p* < 0.001). **(B)** The median (IQR) urine SG levels in anti-NMDAR antibody encephalitis patients with anti-NMDAR antibody <1:32 and anti-NMDAR antibody ≥1:32 were significantly lower than HCs, respectively (*p* = 0.006 and *p* < 0.001). * represents significant difference between the two groups, *p* < 0.05.

The unadjusted median and interquartile range (IQR) urine pH levels in patients with anti-NMDAR antibody <1:32 vs. patients with anti-NMDAR antibody ≥1:32 were 6.75 (6.00–7.00) vs. 7.00 (6.50–7.00), *p* = 0.029 (Table 2; Figure 2A). And the median (IQR) urine pH levels in HCs were 6.00 (5.50–6.50). The unadjusted median and interquartile range (IQR) urine pH levels in anti-NMDAR antibody encephalitis patients with anti-NMDAR antibody <1:32 and anti-NMDAR antibody ≥1:32 were significantly higher than in HCs, respectively (both *p* < 0.001; Table 2; Figure 2A). The median (IQR) urine SG levels in anti-NMDAR antibody encephalitis patients with anti-NMDAR antibody <1:32 and anti-NMDAR antibody ≥1:32 were significantly lower than in HCs, (*p* = 0.006 and *p* < 0.001; Table 2; Figure 2B).

In addition, we also divided these patients into two groups according to mRS (0–2 and 3–5), pH (pH ≤ 6.5 and pH > 6.5), and USG (≤ 1.015 and > 1.015). These results were shown in Supplementary Tables 1–3.

### Correlation Analysis in Anti-NMDAR Antibody Encephalitis Patients at Initial Admission

**Figure 3:**
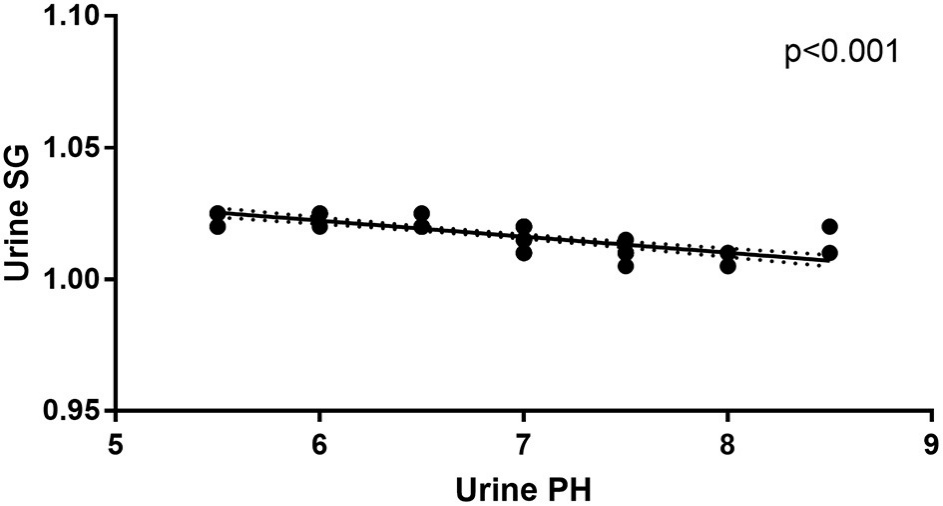
Correlation between urine pH and urine SG levels in the anti-NMDAR antibody patients at initial admission.

In addition to urine SG levels correlating with urine pH levels in anti-NMDAR antibody encephalitis patients at initial admission (*r* = −0.006, *p* < 0.001; Figure 3), there were no correlations between mRS scores, disease duration, Scr levels, urine SG levels, or urine pH levels. And there were no correlations between mRS scores, disease duration, urine SG levels, or urine pH levels and eGFR levels from anti-NMDAR antibody encephalitis patients at initial admission.

### Follow-Up Evaluation in Anti-NMDAR Antibody Encephalitis Patients Following Treatments

**Figure 4:**
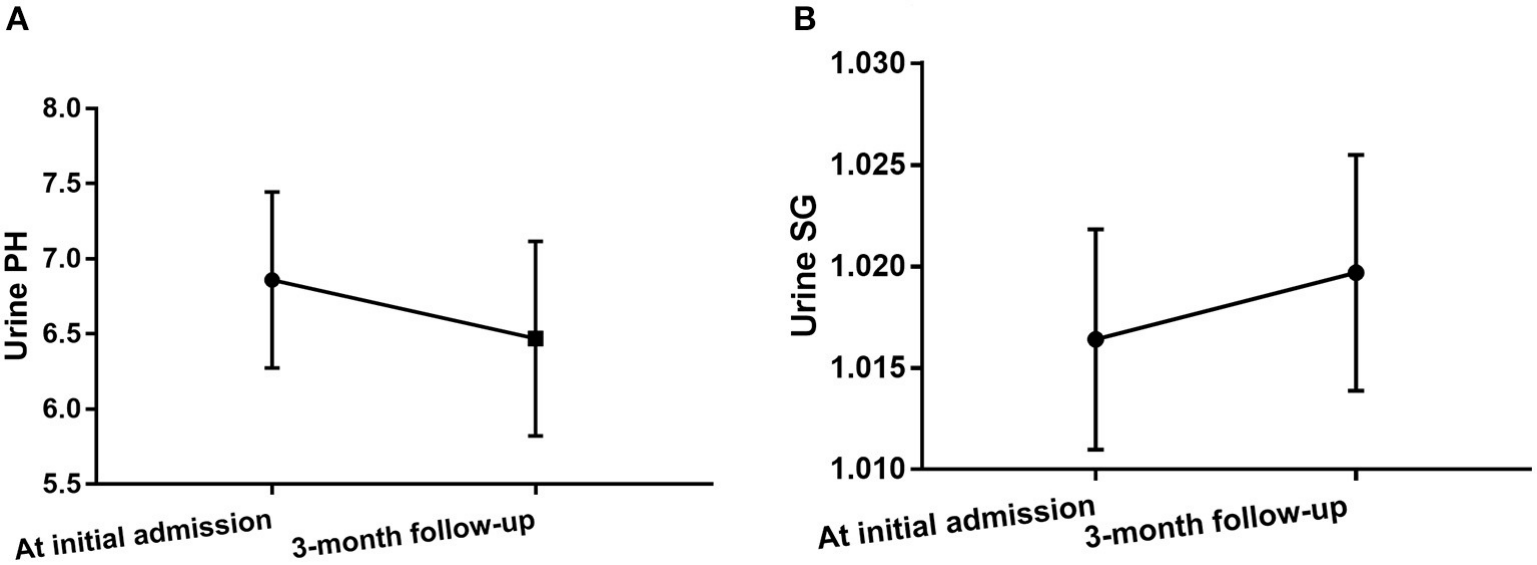
Changes in urine pH levels **(A)** and urine SG levels **(B)** in anti-NMDAR antibody encephalitis patients after treatment.

Urine pH levels were significantly lower in follow-up evaluation 3 months after treatment than at initial admission (*p* = 0.004), while urine SG levels were higher in follow-up evaluation 3 months after treatment than at initial admission (*p* = 0.027), as shown in Table 3, Figure 4, Supplementary Figure 1. Furthermore, mRS scores were significantly lower in follow-up evaluation 3 months after treatment than at initial admission. There were no differences in Scr or eGFR levels in follow-up evaluations 3 months after treatment and at initial admission.

**Table 3:** 3-month follow-up evaluation in anti-NMDAR antibody encephalitis patients.

	**Anti-NMDAR antibody encephalitis (*****n*** **=** **32)**		
	**At initial admission**	**3–month follow-up**	***p-*value**
Sex (male: female)	18:14	18:14	1.000^P1^
Scr levels (μmol/L, IQR)	60.50 (46.00–77.50)	61.50 (49.25–77.75)	0.456^P2^
eGFR levels (mL/min/1.73 m2, IQR)	122.25 (107.48–136.29)	120.27 (109.20–131.25)	0.636^P2^
urine pH levels	7.00 (6.50–7.00)	6.50 (6.00–7.00)	0.004^P2^
urine SG levels	1.015 (1.010–1.020)	1.020 (1.015–1.025)	0.027^P2^
mRS	3.00 (1.25–4.00)	1.00 (0.00–2.00)	<0.001^P2^

*anti-NMDAR, anti-N-Methyl-D-aspartate receptor; Ab, antibody; Scr, Serum Creatinine; GFR, glomerular filtration rate; eGFR, estimated GFR; SG, Specific Gravity; SD, standard deviation; IQR, interquartile range. P1, Chi-square test; P2, paired Mann-Whitney U-tests*.

## Discussion

To the best of our knowledge, this study is the first to analyze the results from urinalysis and renal function in patients with anti-NMDAR antibody encephalitis. In this study, we found that urine pH and urine SG levels in anti-NMDAR antibody encephalitis patients at initial admission were significantly higher and lower than HCs, respectively. Urine pH levels in patients with anti-NMDAR antibody <1:32 were significantly lower than patients with anti-NMDAR antibody ≥1:32. The median urine pH levels and urine SG levels in anti-NMDAR antibody encephalitis patients at initial admission were higher and lower than those in follow-up evaluation 3 months after treatment, respectively.

Microarray studies have shown that all known NMDAR transcripts can be detected in the kidney (5), such as GluN1, GluN2A, C, D, GluN3A, and B. NMDAR-mediated effects of both have been observed in the kidney. The proposed functions are vasodilation, proximal reabsorption, glomerular filtration, and renotoxicity (15–19). NMDARs are expressed in the renal cortex and medulla and appear to play a role in the regulation of renal blood flow, glomerular filtration, proximal tubule reabsorption, and urine concentration within medullary collecting ducts (6). A study has found that NR3a is localized to the basolateral membrane of the collecting duct in the kidney, which may play a renoprotective role in collecting duct cells (17). Urine SG is performed to evaluate the kidney's ability to dilute or concentrate urine in order to maintain homeostasis (20). The affected organs outside of CNS in anti-NMDAR antibody encephalitis, such as the lymphoid tissue, testicular tissue, and even kidney, have been described in several previous reports and may have been involved (21–23). In this study, we found urine SG levels were significantly lower than HCs in anti-NMDAR antibody encephalitis patients at initial admission. This finding suggested that anti-NMDAR antibody encephalitis patients may have defects in their ability to concentrate urine. Furthermore, the collecting duct is also responsible for acid/base transport (24). We found urine pH levels in anti-NMDAR antibody encephalitis patients at initial admission were significantly higher than HCs. The results suggested urine pH levels in anti-NMDAR antibody encephalitis patients are more likely to be alkaline than HCs. We further found different antibody titers had effects on urine pH. Compared with patients with anti-NMDAR antibody ≥1:32, urine pH in patients with anti-NMDAR antibody <1:32 was closer to the HCs. Therefore, our study suggested that the antibody against NMDAR has an impact on the collecting duct. We further analyzed the effect on the results of urinalysis in follow-up evaluation 3 months after treatment; we found with the improvement of the disease condition, the urine pH levels decreased and the urine SG levels increased. According to our results, the effect on the kidney from anti-NMDAR antibody encephalitis is involved in impaired urine concentration and changes to urine pH and urine SG were observed to be a marker of the improvement of anti-NMDAR antibody encephalitis.

In this study, we found there were no significant differences in Scr and eGFR between anti-NMDAR antibody encephalitis patients and HCs. According to this result, our study suggested there were no negative effects on renal function indexes, such as Scr and eGFR, in anti-NMDAR antibody encephalitis. There is now a consensus that activation of the NMDA receptor affects renal function, and in some cases may induce renal dysfunction (6). Similarly, pharmacological inhibition of NMDARs in proximal tubules ameliorated renal insufficiency in an animal model of acute kidney injury (19). Also in animal models, ischemia results in the upregulation of NR1 subunits throughout the kidney, and NMDA blockade is reported to improve renal function after ischemia (25). As mentioned above, antibodies against NMDAR may not have a deteriorating effect on renal function indexes such as Scr and eGFR in patients with anti-NMDAR antibody encephalitis. However, there is a study analyzing autopsy data of one male patient with anti-NMDAR antibody encephalitis, which found renal edema with renal failure due to acute tubular necrosis (23). This may be explained by the fact that the above patient was deceased, which may eventually lead to kidney damage.

However, there are limitations to this study. Firstly, this is a retrospective study on only a single ethnic population from a single center, which could result in unintentional bias. Secondly, the numbers of patients were relatively small, especially the numbers of patients at initial admission. However, to the best of our knowledge, this is the first study to evaluate urinalysis and renal function in anti-NMDAR antibody encephalitis. Anti-NMDAR antibody encephalitis is a rare neuroimmunological disease of CNS. In the future, we will collect more patients for further investigation. Thirdly, we did not use eGFR_CysC_ and eGFR_Cr−CysC_ to evaluate renal function in this study. The estimated GFR (eGFR) used in this study is Cr-based eGFR, which can be affected by a patient's age, gender, race, weight, height, body surface area, and particularly by muscle mass and dietary intake. eGFR_CysC_ or eGFR_Cr−CysC_ could provide a more accurate measure of renal function. The measurement of eGFR_CysC_ or eGFR_Cr−CysC_ may improve the sensitivity and specificity of the assessment of kidney function, which will be investigated in the future.

In conclusion, our results indicate that urine pH levels and urine SG levels in anti-NMDAR antibody encephalitis patients were significantly higher and lower than HCs, respectively. These findings suggest pathophysiological changes in anti-NMDAR antibody encephalitis patients involve not only the central nervous system, but also the kidney, especially the collecting duct.

## Data Availability Statement

The raw data supporting the conclusions of this article will be made available by the authors, without undue reservation.

## Ethics Statement

The studies involving human participants were reviewed and approved by the Medical Ethics Committee of the Third Affiliated Hospital of Sun Yat-sen University. The patients/participants provided their written informed consent to participate in this study.

## Author Contributions

YJ and FP contributed to the conception and design of this study. LL, MG, JL, QL, XX, and RF collected and organized the data. LL, MG, JL, QL, and YJ analyzed the data. YJ, FP, LL, MG, JL, QL, and RF drafted the manuscript. All authors read and approved the final manuscript.

## Conflict of Interest

The authors declare that the research was conducted in the absence of any commercial or financial relationships that could be construed as a potential conflict of interest.

## Supplementary Material

Supplementary Figure 1 **(A)** Urine SG levels of the same anti-NMDAR antibody encephalitis patients at initial admission and in a follow-up evaluation 3 months after treatment. **(B)** Urine pH levels of the same anti-NMDAR antibody encephalitis patients at initial admission and in a follow-up evaluation 3 months after treatment.

Supplementary Table 1 Comparison between mRS 0–2 and mRS 3–5 in anti-NMDAR antibody encephalitis patients at initial admission.

Supplementary Table 2 Comparison between pH ≤ 6.5 and pH > 6.5 in anti-NMDAR antibody encephalitis patients at initial admission.

Supplementary Table 3 Comparison between USG ≤ 1.015 and USG > 1.015 in anti-NMDAR antibody encephalitis patients at initial admission.
