# Supplementary figures and images for: The Effect on the Kidney in Patients With Anti-N-methyl D-aspartate Receptor Antibody Encephalitis
Source: Front Neurol. 2021 Feb 12;12:601495. doi: 10.3389/fneur.2021.601495 (PMC7907499; doi:10.3389/fneur.2021.601495)

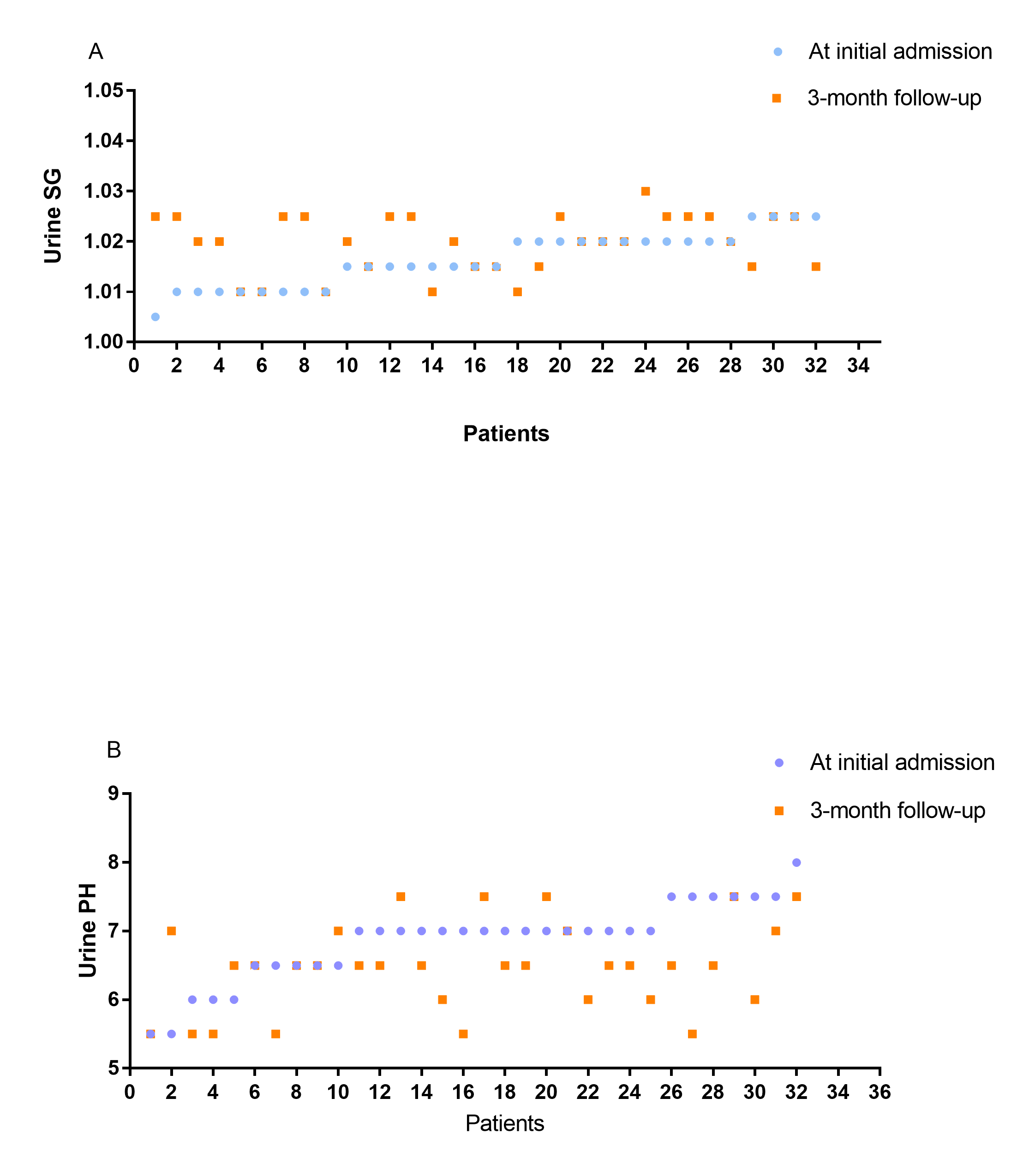

Supplement: Supplementary Figure 1 — (A) Urine SG levels of the same anti-NMDAR antibody encephalitis patients at initial admission and in a follow-up evaluation 3 months after treatment. (B) Urine pH levels of the same anti-NMDAR antibody encephalitis patients at initial admission and in a follow-up evaluation 3 months after treatment. [file Image_1.TIF]
