# Supplementary material for: The Effect on the Kidney in Patients With Anti-N-methyl D-aspartate Receptor Antibody Encephalitis
Source: Front Neurol. 2021 Feb 12;12:601495. doi: 10.3389/fneur.2021.601495 (PMC7907499; doi:10.3389/fneur.2021.601495)
Supplement: Supplementary Table 1 — Comparison between mRS 0–2 and mRS 3–5 in anti-NMDAR antibody encephalitis patients at initial admission. [file Table_1.docx]

**Table S1. Comparison between** **mRS 0-2 and mRS 3-5 in anti-NMDAR antibody encephalitis patients at initial admission**

|  |  | **mRS 0-2** |  | **mRS 3-5** |  |  |
| --- | --- | --- | --- | --- | --- | --- |
| Variables |  | (n =28) |  | (n =54) |  | p value |
| **Age onset (y, mean±SD)** |  | 31.04±13.21 |  | 32.67±11.84 |  | 0.571^P1^ |
| **Sex, male: female** |  | 12: 16 |  | 25: 29 |  | 0.190^P3^ |
| **Disease duration (d, IQR)** |  | 22.00(13.00-27.00) |  | 27.50(19.75-40.50) |  | 0.022^P2^ |
| **Scr (umol/L, IQR)** |  | 61.00(48.00-71.50) |  | 56.00(46.50-76.50) |  | 0.922^P2^ |
| **eGFR (ml/(min×1.73m2), IQR)** |  | 122.97(109.25-133.56) |  | 123.95(108.27-133.51) |  | 0.826^P2^ |
| **Urine pH levels** |  | 7.00(6.50-7.00)^a^ |  | 7.00(6.50-7.00)^b^ |  | 0.861^P2^ |
| **Urine SG levels** |  | 1.020(1.010-1.020)^a^ |  | 1.020(1.015-1.020)^b^ |  | 0.574^P2^ |

Note: anti-NMDAR, anti-N-Methyl-D-aspartate receptor; Scr, Serum Creatinine; GFR, glomerular filtration rate; eGFR, estimated GFR; SG, Specific Gravity; SD, standard deviation; IQR, interquartile range. a. N=26; b, N=53, p1, the Student’s t test; p2, Mann-Whitney U tests; p3, Chi-square test.
