# Supplementary material for: The Effect on the Kidney in Patients With Anti-N-methyl D-aspartate Receptor Antibody Encephalitis
Source: Front Neurol. 2021 Feb 12;12:601495. doi: 10.3389/fneur.2021.601495 (PMC7907499; doi:10.3389/fneur.2021.601495)
Supplement: Supplementary Table 2 — Comparison between pH ≤ 6.5 and pH > 6.5 in anti-NMDAR antibody encephalitis patients at initial admission. [file Table_2.docx]

**Table S2. Comparison between pH *≤* 6.5 and pH > 6.5 in anti-NMDAR antibody encephalitis patients at initial admission**

|  |  | **PH ≤ 6.5** |  | **PH > 6.5** |  |  |
| --- | --- | --- | --- | --- | --- | --- |
| Variables |  | (n =30) |  | (n =49) |  | p value |
| **Age onset (y, mean±SD)** |  | 31.50±12.72 |  | 32.84±12.18 |  | 0.643^P1^ |
| **Sex, male: female** |  | 12: 18 |  | 25: 24 |  | 0.341^P3^ |
| **Disease duration (d, IQR)** |  | 26.50(20.00-36.25) |  | 24.00(15.00-34.50) |  | 0.302^P2^ |
| **mRS (IQR)** |  | 3.50(2.00-5.00) |  | 4.00(1.00-4.50) |  | 0.668^P2^ |
| **Cr (umol/L, IQR)** |  | 57.00(50.00-78.25) |  | 58.00(44.50-73.00) |  | 0.682^P2^ |
| **eGFR (ml/(min×1.73m^2^), IQR)** |  | 121.37(109.06-132.00) |  | 123.43(107.85-133.68) |  | 0.932^P2^ |
| **Urine SG** |  | 1.020(1.020-1.025) |  | 1.015(1.010-1.020) |  | <0.001^P2^ |

Note: anti-NMDAR, anti-N-Methyl-D-aspartate receptor; Scr, Serum Creatinine; GFR, glomerular filtration rate; eGFR, estimated GFR; SG, Specific Gravity; SD, standard deviation; IQR, interquartile range. P1, the Student’s t test; p2, Mann-Whitney U tests; p3, Chi-square test.
