# Supplementary material for: The Effect on the Kidney in Patients With Anti-N-methyl D-aspartate Receptor Antibody Encephalitis
Source: Front Neurol. 2021 Feb 12;12:601495. doi: 10.3389/fneur.2021.601495 (PMC7907499; doi:10.3389/fneur.2021.601495)
Supplement: Supplementary Table 3 — Comparison between USG ≤ 1.015 and USG > 1.015 in anti-NMDAR antibody encephalitis patients at initial admission. [file Table_3.docx]

**Table S3. Comparison between USG ≤ 1.015 and USG > 1.015 in anti-NMDAR antibody encephalitis patients at initial admission**

|  |  | **USG ≤ 1.015** |  | **USG > 1.015** |  |  |
| --- | --- | --- | --- | --- | --- | --- |
| Variables |  | (n =34) |  | (n =45) |  | p value |
| **Age onset (y, mean±SD)** |  | 32.26±13.10 |  | 32.38±11.85 |  | 0.968^P1^ |
| **Sex, male: female** |  | 16: 18 |  | 21: 24 |  | 0.972^P3^ |
| **Seizure (n, %)** |  | 19 (55.88) |  | 27 (60.00) |  | 0.713^P3^ |
| **Disease duration (d, IQR)** |  | 26.00(18.25-34.25 |  | 24.00(15.50-35.00) |  | 0.736^P2^ |
| **mRS(IQR)** |  | 4.00(1.00-5.00) |  | 3.00(2.00-5.00) |  | 0.980^P2^ |
| **Cr (umol/L, IQR)** |  | 59.50(44.00-72.00) |  | 58.00(49.00-78.50) |  | 0.569^P2^ |
| **eGFR (ml/(min×1.73m2), IQR)** |  | 126.59(108.62-137.31) |  | 120.23(107.16-131.49) |  | 0.549^P2^ |
| **pH** |  | 7.00(7.00-7.50) |  | 6.50(6.00-7.00) |  | <0.001^P2^ |

Note: anti-NMDAR, anti-N-Methyl-D-aspartate receptor; Scr, Serum Creatinine; GFR, glomerular filtration rate; eGFR, estimated GFR; SG, Specific Gravity; SD, standard deviation; IQR, interquartile range. P1, the Student’s t test; p2, Mann-Whitney U tests; p3, Chi-square test.
